# The Dental Department of Iowa University

**Published:** 1889-04

**Authors:** 


### Editorial, Etc.

The Dental Department of Iowa University.—The legislative investing committee report of affairs in the Iowa state university is a voluminous document, but its ponderousness does not entirely conceal some interesting facts. A synopsis of its general scope has already been made public, and I have obtained an advance copy of that section of the report relating to the dental department which brings to light some interesting facts not heretofore generally known. It reveals some surprising mismanagement—to put it mildly—in the dental department, and while it is a side issue to the main question which led to the appointment of a committee to investigate the university's affairs, it alone justifies the appointment and work of the committee. There is much to be read "between lines" and I am told there has been a good deal of a "circus" going on at Iowa City not yet made public, and that the band is still playing. For the present, however, I will let the committee speak for itself.

This department was organized by the board of regents in 1882 and with the understanding with the dental profession of the state that it should be run without expense to the state until such time as the state should recognize it by making an appropriation for its support.

In 1884 the twentieth general assembly made an appropriation of five hundred dollars to assist in equipping the department with illustrative apparatus, dental chairs and furniture for the lecture room.

This it was claimed by the dental professors was a recognition of their department by the state, and they asked to be fully adopted into the family of the university, with all the rights and privileges of the other departments of the university including the payment of salaries to the professors.

Their prayer was not granted, or at least only conditionally, and the twenty-first general assembly was beseiged for an appropriation to pay salaries. This was denied, however an appropriation of two thousand dollars was made for the further equipment of the department. The professors renewed their petition and asked for the payment of back salaries. The board then formally adopted them, but paid back salaries for two years only. This was the recognition sought to be obtained by an appropriation from the state, and for which the state paid to one of the professors over eight hundred dollars as a lobbyist in addition to his salary as a professor.

For the first two years it was run without expense to the state, and the state is not particularly interested in its management, but since 1884 it has to all intents and purposes been a state institution, and as such we do not hesitate to say its management has been most execrable.

Although the university could have purchased everything needed for its equipment at a discount of from 15 to 25 per cent. from wholesale prices, the professors were allowed to turn over to the university a lot of second-hand instruments and apparatus at full retail prices, although they being connected with the university had bought them at the discount named.

It is but fair to state, however, that Professors Ingersoll and Wilson claim to have objected to this method of speculating.

Professor Hunt seems to have had, except the last year, the entire management of the finances of the department. He has purchased supplies and collected the fees. He has kept the supplies for the department in his own office and borrowed from them when out of material and at the same time run a sort of supply department for the students. He has banked university money with his own bank account—he has been secretary, treasurer, book-keeper, and general manager and as such has had entire charge and control of all the books and papers of the department.

For these things he has been severely criticised by his colleagues, and by implication at least charged with appropriating department material and funds to his own account. We are of the opinion that Dr. Hunt is not the only one deserving of censure, the other members of the faculty and board of regents were to blame for permitting this condition of things to exist at all.

Dr. Hunt presented his books and vouchers for our inspection and we found no evidence of anything wrong with them except that they were meager and failed to make a complete exhibit of the affairs of the departments. Neither did his colleagues, Professors Ingersoll and Wilson; although they evidently expected so to do, disclose any discrepancies.

During the last session the board of regents assumed the entire financial management of the department and the clinical receipts more than doubled those of the last session previous proving to us conclusively that while there may not have been any diversion of funds there must have been great mismanagement. But our criticism does not end here, for we find that by a recent action of the board Professors Ingersoll and Wilson, against whom no breath of suspicion has been raised, no word of complaint entered, have been removed from the faculty, while Professor Hunt, who has at least the smell of fire upon his garments, has been retained and advanced to the position of dean of the faculty, and this in the full light of the developments of this investigation, and in the face of a series of resolutions signed by one-half of the junior members of the last class declaring that he had been neglectful of his duties to the department and asking for his removal. We are credibly informed that but few of the sixteen students signing these resolutions have returned this year. When we consider these facts the action of the board is unaccountable. They may have good and sufficient reasons for their action, but they have not seen fit to make them known.

Since appointed to our present duty we have made a careful study of the university and we believe we have a very fair understanding of its needs, and we cannot conceive of any exigency which would make it necessary to remove two such men as Professors Ingersoll and Wilson and retain Professor Hunt for the good of the university.

We are told that the department is prosperous, having more students in attendance this session than ever before. We are glad to know of its prosperity, but to our minds it only proves the popular demand for such instruction, and that the department can prosper in spite of its mismanagement.—*Burlington Hawkeye.*

